# Disease duration, age at diagnosis and organ damage are important factors for cardiovascular disease in SLE

**DOI:** 10.1136/lupus-2020-000398

**Published:** 2020-06-24

**Authors:** Ola Nived, Ragnar Freyr Ingvarsson, Anna Jöud, Petrus Linge, Helena Tydén, Andreas Jönsen, Anders A Bengtsson

**Affiliations:** 1 Department of Rheumatology, Institution for Clinical Sciences, Lund, Sweden; 2 Landspitali University Hospital, Reykjavik, Iceland; 3 Division of Occupational and Environmental Medicine, Department of Laboratory Medicine, Lund University, Lund, Sweden

**Keywords:** lupus erythematosus, systemic, cardiovascular diseases, epidemiology

## Abstract

**Objective:**

To report the incidence rate ratios (IRR) of acute myocardial infarctions (AMI) and cerebrovascular events (CVE) in incident SLE cases from a defined population. To study the risk factors for cardiovascular events in all patients with SLE at our unit.

**Methods:**

Patients with SLE diagnosed from 1981 to 2006 were followed through to 2016. IRRs of AMI and CVE were calculated. The AMI and CVE incidence patterns for patients with SLE were studied in relation to hypertension, smoking, renal dysfunction, anticardiolipin (aCL) antibodies at diagnosis, disease duration and organ damage before an event.

**Results:**

262 patients with SLE were included in the study; of these 175 were from the defined population. Overall, 37 AMI and 44 CVE were recorded. An increased IRR of 3 for AMI was found (p<0.001). Smoking, hypertension and reduced renal function were risk factors for AMI. An increased IRR of 3.3 for ischaemic CVE was found for women (p<0.001). Hypertension and aCL were risk factors for CVE. Organ damage before events was increased.

**Conclusions:**

Cardiovascular events are increased in SLE and are associated with hypertension, smoking and increased damage rate.

## Introduction

SLE is an autoimmune disorder with multiple organ manifestations. Morbidity in early disease is characterised by active inflammatory processes, while later morbidity is primarily as a consequence of long-standing inflammation or treatment with corticosteroids leading to organ damage, especially cardiovascular disease (CVD). The same bimodal pattern was described in the 1970s for mortality, with active disease or infections involved with shorter disease duration and acute myocardial infarctions (AMI) and possibly cerebrovascular events (CVE) after longer disease duration.^1 2^ With better understanding of the disease mechanisms and more judicious use of different therapeutic modalities these patterns of manifestations might have changed as some reports indicate.^3^ However, a meta-analysis of mortality in SLE indicates that cardiovascular issues remain problematic.^4^ The morbidity for AMI has been shown to be increased with the range 2–50 times in reports from many countries, but with different study populations and stratifications.^5–8^ Many have also reported an increased frequency of CVE in SLE, and recent studies indicate that this is often an early manifestation, and part of the antiphospholipid syndrome.^9–12^ Contrary to previous literature, also AMI has now been reported to occur early after diagnosis of SLE, or in some cases even before diagnosis.^11 13^ Regarding CVD in SLE the most striking finding has been the relatively higher frequency of this manifestation in women before menopause, a group in the general population which often is considered to be protected.^5 8 14^

The proposed risk factors responsible for the observed increase of CVD in SLE are both standard risk factors such as hypertension, hyperlipidaemia, smoking and diabetes,^8 15–17^ and SLE specific. Among the proposed SLE-specific risk factors are disease activity, glucocorticoid use, cytostatic treatment, lupus nephritis, SLE disease duration and antiphospholipid antibodies.^14–20^ It has also been reported that SLE-related factors are seen early in the disease course while traditional risk factors accrue over time.^21^

The aim of this series was to study the frequencies of myocardial infarction and CVEs in patients diagnosed with SLE within a defined population. In addition, we wanted to analyse the cardiovascular events in relation to age at diagnosis, disease duration, accumulated damage before the event and any relationship with the risk factors hypertension and smoking. For risk factor analysis our entire cohort, also including referred patients with SLE from areas outside the local area, was used.

## Materials and methods

### Population under study

A population-based SLE cohort study was initiated in 1981 including all known incident SLE cases in a defined local catchment area of eight counties, with an average population of 196 000 individuals, and surrounding the city of Lund. The completeness of case retrieval in this area has been verified by capture-recapture technique from multiple sources.^22^ The cohort is continuously collected and patients are followed prospectively at the Skåne University Hospital, Unit of Rheumatology. This well-defined cohort was used for calculation of rate ratios of cardiovascular events compared with the general population of the same area, stratified for sex and age. For all other analyses, where a population comparator was not included, also SLE cases from outside the defined area were included. On each case American College of Rheumatology (ACR) classification criteria for SLE,^23–25^ IgG anticardiolipin antibodies, presence of the standard risk factors hypertension, smoking and diabetes, all at diagnosis, were registered. Organ damage has been registered each year up to event (AMI or CVE) with Systemic Lupus International Collaborating Clinics (SLICC)/ACR damage index (DI).^26^ For comparison of organ damage up to event each SLE case with event was matched for sex, age at diagnosis and disease duration, with two patients with SLE without event. When this study was planned, four decades ago, no patient partnership was included.

### Data retrieval

The responsibility for healthcare in Sweden is decentralised to different regions and their regional county councils. Both public and private healthcare is available where the only difference lies in the organisation; all healthcare in Sweden is funded through taxes and the same costs apply to the individual regardless whether the care is provided from a public institution or from a private healthcare provider. In the Region of Skåne, the southernmost part of Sweden, electronic healthcare information is available since 1998 including all hospitalisations for the population, and all visits at both hospital clinics and in primary care. This database, the Skåne Healthcare Register, contains information on International Classification of Diseases (ICD) codes for each contact.

Only incident cases with a new diagnosis of SLE between 1 January 1981 and 31 December 2006 were included. In all, 262 patients with SLE were included in the study (175 patients with SLE from the local cohort with a mean annual prevalence of 115 patients in the defined area). Since only incident cases after 1981 were included, the annual prevalence increased and reached a steady state in 1998 in the defined area, a prerequisite for calculation of rate ratios. (online supplementary figure 1). Only two known patients with SLE in the area refused to participate in our clinic, but we were allowed to follow their data in the registries, so they were not lost to follow-up.

10.1136/lupus-2020-000398.supp1 Supplementary data

The ICD codes in the ICD 10th Revision chapter for cardiovascular disorders were extracted for all patients with SLE with events during the period 1 January 1998 to 31 December 2016 and the codes I21 (AMI), I60, I61, I62, I63 and I64 (all CVEs) were analysed, taken into account only the first AMI and/or CVE diagnosis for each patient. Only events after the diagnosis of SLE were included, and in fact no events occurred before diagnosis of SLE. Events from the time period 1981–1997 were traced in the medical records. If a patient has had both an AMI and a CVE the first event of both categories is included in the analysis. The frequencies of the same diagnoses in the population of the defined area during the same period, stratified for sex, age and calendar year, were also extracted and used for calculation of rates and rate ratios.

In addition, medical records for all patients with SLE were scrutinised for verification of SLE diagnosis, defined previously,^14^ and for cardiovascular events, defined as patients fulfilling the SLICC/ACR-DI.^26^ Hypertension was defined as a systolic blood pressure >140 mm Hg and/or a diastolic blood pressure >90 mm Hg and/or present treatment for hypertension. Smoking data were recorded as ever smoking before event. Reduced glomerular filtration rate before event was defined by the SLICC/ACR-DI. Obesity and dyslipidaemia were not included in the analysis since they were only registered from 1990, why data from time of diagnosis were not available in cases diagnosed earlier. Follow-up ended 31 December 2016 or earlier if the patient was deceased, moved out of the area or was lost to follow-up.

### Statistical analysis

Incidence rates of AMI and CVE per 1000 person-years were calculated. Age and sex-standardised rate ratios with 95% CIs were calculated comparing SLE and non-SLE in the defined area under study. Associations with risk factors, and disease manifestations were determined with non-parametric statistics, Mann-Whitney U-test was used when comparing groups including matched pairs, or parametric statistics, Student’s t-test, when appropriate. For the matched pairs, when analysing the frequency of damage before event, a patient with SLE with an event was matched with one or two patients with SLE without the event for the same age at diagnosis (±3 years), same sex and same disease duration. A significance level of 0.05 was applied if nothing else is stated. Analysis was done using Statistica V.6.0. We used multivariable Cox regression to examine the possible influence of different covariates, other than the accumulated damage described above, on time to cardiovascular events. These statistical calculations were performed using RStudio V.1.1.456.

## Results

In total, 262 patients with SLE diagnosed between 1981 and 2006 were included. Of these, 175 lived in the defined catchment area of the local cohort. In the local cohort the prevalence for SLE was stable during the period 1998–2016 with a mean annual number of 115 cases in the defined area, or 59 SLE cases per 100 000 individuals. For women, the prevalence was 101 per 100 000 women. Patient and population demographic data and potential risk factors for CVD present at diagnosis are given in tables 1a and b. All patients in the defined area were Caucasian except one of Asian origin. No cases were lost to follow-up.

**Table 1a:** Demographic data and risk factors in the total SLE cohort

Variable	Patients with SLE
n total	262
Females	224 (86%)
Mean age (range)	43.4 years (10–88)
Hypertension	39/262 (15%)
Smoking ever (before event)	71/118 (60%)
Diabetes	1/262

**Table 1b:** Demographic data in the SLE cohort from the defined area

Variable	SLE-defined area	Mean population-defined area (1998–2007)
n total	175	196 259 (age >17 years)
Females	149 (85%)	99 245 (51%)
Mean annual prevalence (cases/100 000 individuals)	59	
Females	101 (88%)	
Mean age (range)	48.3 years (15–88)	40.3 years (0 to 100+)

In total, 37 AMI cases among patients with SLE were registered. Of these, 16 occurred in the defined cohort during the period 1998–2016 and were used for calculation of rate ratios. There were 44 cases of CVE among the patients with SLE and of these 21 occurred in the defined cohort between 1998 and 2016. Both AMI and CVE were registered in 10 cases, six of these in the defined cohort. In seven of these cases, with both CVE and AMI, the CVE preceded the AMI by at least 2 years (range 2–26 years) and for the remaining three cases, with the AMI first, the time difference was at least 4 years (range 4–9 years). In total, there were 81 first events in 71 patients with SLE. The total follow-up time was 5560 patient-years, of these 3553 patient-years were in the cohort from the defined area with median disease duration and also mean duration from inclusion of 19 years (range 1–36 years, SD=8.7).

The incidence rate ratios (IRR) for first myocardial infarction (AMI) are given in table 2. In summary, the IRR for AMI was 3.0 in SLE (95% CI 1.3 to 6.9, p<0001), and the increased rate was most pronounced for women below 60 years of age. An increased rate was only seen in males in the age group 40–59 years where the increased rate was 7.7 (95% CI 1.5 to 39.9, p<0.001). There were no significant increased rates in ages 60 years or older, for neither women nor for men.

**Table 2:** Incidence rate ratio for myocardial infarction in SLE 1998–2016

Age at event	All ages		Age <40 years		Ages 40–59 years		Age ≥60 years	
	SLE	Non-SLE	SLE	Non-SLE	SLE	Non-SLE	SLE	Non-SLE
**Both sexes**								
n (mean prevalence)	115	196 259	18	77 294	40	65 211	57	53 754
Events	16	9051	1	66	3	1256	12	7729
Incidence ratio/1000 patient-years	7.3	2.4	2.9	0.9	3.9	1.0	11.1	7.6
Crude IRR	3.0		3.2		3.9		1.5	
CI (95/99.9%) P value	1.3 to 6.9 <0.001		1.5 to 7.0 <0.001		1.3 to 12.1 <0.05		0.85 to 2.6 n.s.	
**Females**								
n	101	99 425	15	37 806	36	32 535	50	29 084
Events	14	3629	1	15	2	281	11	3333
IR/1000 patient-years	7.3	1.9	3.5	0.02	2.9	0.5	11.6	6.0
Crude IRR	3.8		175		5.8		1.9	
CI (99/99.9%) P value	1.6 to 9.3 <0.001		74.8 to 409 <0.001		1.1 to 39.5 <0.01		0.3 to 12.3 n.s.	
**Males**								
n	14	96 834	3	39 488	4	32 676	7	24 670
Events	2	5422	0	51	1	975	1	4396
IR/1000 patient-years	7.5	2.9	–	–	12.1	1.6	7.52	9.4
Crude IRR	2.6		–		7.7		0.8	
CI (99.9%) P value	0.7 to 10.3 n.s.		–		1.5 to 39.9 <0.001		0.1 to 5.7 n.s.	

IR, incidence rate; IRR, incidence rate ratio; n.s., not significant.

**Figure 1:**
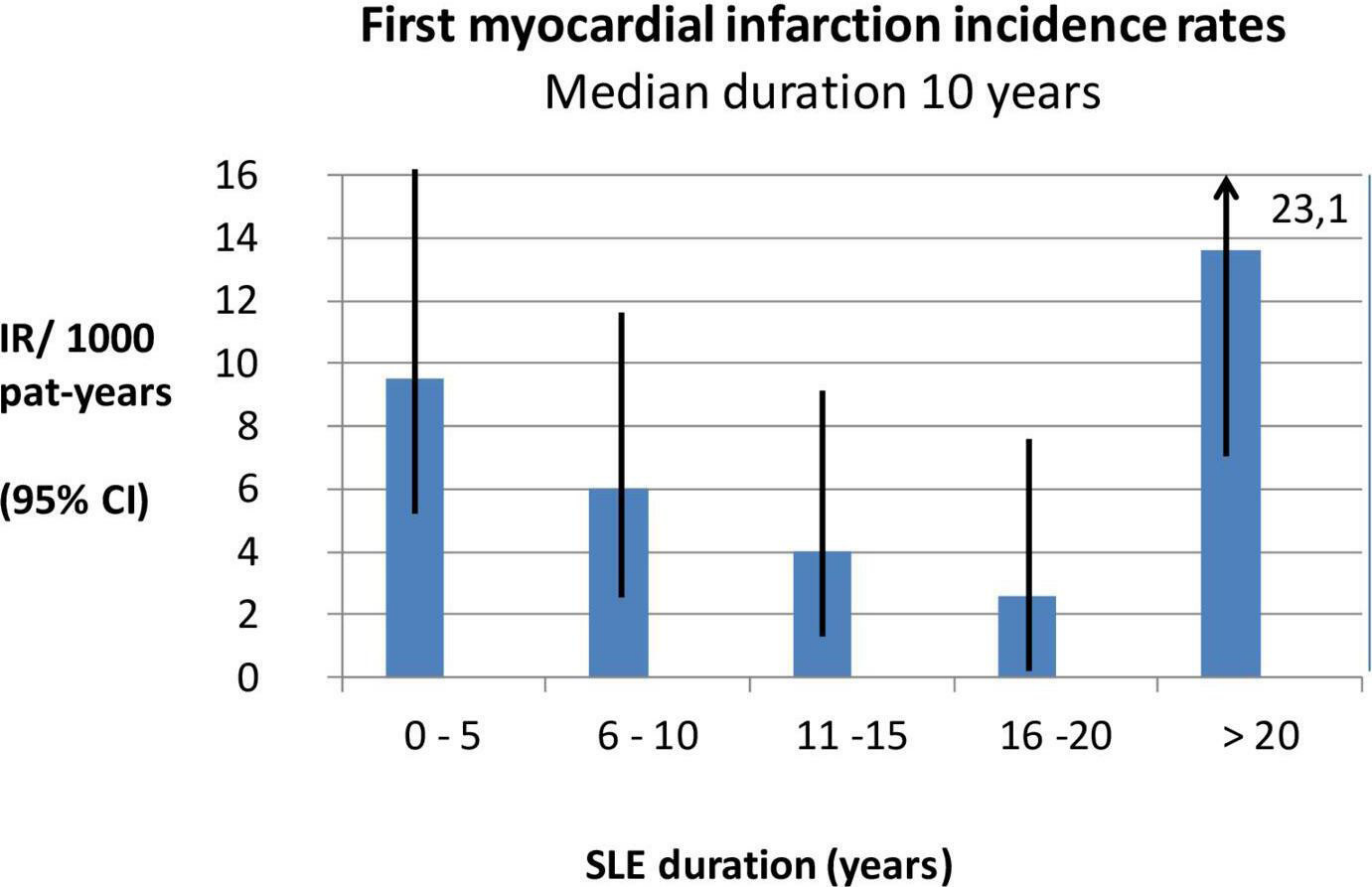
SLE disease duration related to incidence rates for first myocardial infarction in the total SLE cohort and based on patients with an SLE diagnosis between 1981 and 2006 and followed through to 2016 (n=262 patients). IR, incidence rate.

The incidence pattern for myocardial infarction in relation to SLE duration in all patients (n=262) is illustrated in figure 1. The increased incidence of AMI early after diagnosis of SLE was seen in patients with an SLE diagnosis at age of or above 60 years (median duration 4 years, range 0–30 years), while the increased AMI frequency in patients younger than 60 years of age occurred after longer disease duration (median duration 20 years, range 0–32 years). This difference was significant (p<0.01). Premenopausal female patients with SLE younger than 55 years of age had their first AMI after a median disease duration of 20 years, while patients 55 years or older had their first AMI after a median disease duration of only 7 years (p<0.05).

**Figure 2:**
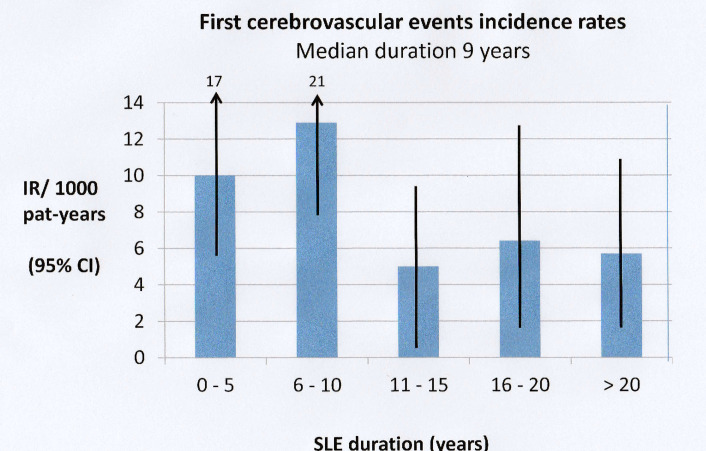
SLE disease duration related to incidence rates for first cerebrovascular event in the total SLE cohort and based on patients with an SLE diagnosis between 1981 and 2006 and followed through to 2016 (n=262 patients). IR, incidence rate.

The IRRs for ischaemic CVE are given in table 3. An IRR of 3.3 for CVE in women with a diagnosis of SLE indicated an increased rate (95% CI 2.4 to 4.6, p<0.001). The incidence pattern for first CVE in relation to SLE duration in all patients is shown in figure 2, and the majority of events occurred during the first 10 years after diagnosis. Of the 44 first CVE, three are registered as subarachnoid bleeding and six as intracerebral bleeding; the remaining 35 events were classified as ischaemic CVE (data not shown).

**Table 3:** Incidence rate ratio for ischaemic cerebrovascular disorder in SLE 1998–2016

Age at event	All ages		Age <40 years		Ages 40–59 years		Age ≥60 years	
	SLE	Non-SLE	SLE	Non-SLE	SLE	Non-SLE	SLE	Non-SLE
**Both sexes**								
n (mean prevalence)	115	196 259	18	77 294	40	65 211	57	53 754
Events	16	8457	0	169	4	910	12	7424
Incidence ratio/1000 patient-years	4.5	2.2	–	–	5.3	0.71	11.0	7.3
Crude IRR	2.1		–		7.5		1.5	
CI (99/99.9%) P value	1.5 to 2.8 <0.001				4.5 to 12.7 <0.001		1.1 to 2.1 <0.01	
**Females**								
n	101	99 425	15	37 806	36	32 535	50	29 084
Events	14	4115	0	56	4	313	10	3746
IR/1000 patient-years	7.3	2.2	–	–	5.8	0.5	10.5	6.8
Crude IRR	3.3		–		11.6		1.5	
CI (95/99.9%) P value	2.4 to 4.6 <0.001				2.0 to 6.1 <0.001		1.1 to 2.0 <0.05	
**Males**								
n	14	96 834	3	39 488	4	32 676	7	24 670
Events	2	4342	0	67	0	597	2	3778
IR/1000 patient-years	7.5	2.4	–	–	–	–	15.0	7.8
Crude IRR	3.1						1.9	
CI (95%) P value	1.8 to 5.2 <0.05						0.9 to 4.0 n.s.	

IR, incidence rate; IRR, incidence rate ratio; n.s., not significant.

Selected risk factors for AMI and CVE, presented at diagnosis of SLE, are listed in table 1. Smoking ever was seen more often in patients who eventually developed AMI (non-parametric statistics, p=0.01). In multivariable regression only gender and hypertension at diagnosis remained in the analysis due to many missing data for smoking. Hypertension was more common both for AMI and CVE as compared with patients without events (tables 4a and b), but for those who developed AMI this was true only for patients with AMI before an age of 55 years (online supplementary figure 2a–d). Mean time to an event was 4.8 years (n=21, SD 5.96) for patients with SLE and hypertension compared with 12.4 years (n=17, SD 11.24) for patients without hypertension (p<0.01).

10.1136/lupus-2020-000398.supp2 Supplementary data

**Table 4a:** Time to acute myocardial infarction in relation to gender and hypertension

	HR	Lower 0.95	Upper 0.95	P value
Multivariate Cox regression				
Gender	2.258	1.021	4.996	0.004*
Hypertension	2.099	1.081	4.078	0.029

*Shorter time to acute myocardial infarction (AMI) for males with SLE.

**Table 4b:** Time to cerebrovascular event in relation to gender and hypertension

	HR	Lower 0.95	Upper 0.95	P value
Multivariate Cox regression				
Gender	0.4878	0.1507	1.579	0.231
Hypertension	2.5878	1.3807	4.85	0.003

Our study indicates that in patients with SLE reduced glomerular filtration rate (<50 mL/min) was associated with AMI (p<0.05). The presence of positive IgG anticardiolipin antibodies was associated with CVE (p<0.05).

Organ damage was measured by SLICC/ACR-DI. In our study, the SLICC/ACR-DI scores were extracted for patients with SLE who subsequently had an event and for two matched patients with SLE without events. The values extracted were 87 points for the 37 AMI cases (median 1) and 100 points for the 73 matched patients with SLE with no AMI (median 1). Similarly, the SLICC/ACR-DI scores before first CVE were 88 points in the 44 cases (median 2) and 70 points in 74 matched patients with SLE with no CVE (median 1). A higher SLICC/ACR damage score was associated with a higher rate of event (AMI p=0.03, CVE p=0.002, Mann-Whitney). Thus, patients with SLE having CVD events had an increased rate of pre-existing irreversible organ damage before the actual event.

## Discussion

In this cohort study analysing the rate of CVD in patients with SLE we confirmed previous findings of increased rates of both AMI and CVE seen in this disorder, especially in women and for AMI also in women before menopause.^5 8 10 12 15 16 18^ Males are few and have only two ischaemic CVE events in the higher age group.

A major strength of this study was the long follow-up since cardiovascular events do not necessarily develop shortly after SLE debut. A second strength was the comprehensive registries available, both for research within our unit and for the healthcare registries in the region with complete coverage since 1998. Another advantage was the population-based approach with all known SLE cases in a defined area collected prospectively since 1981, rendering an expanding cohort, stable from 1998, representative for SLE and thus allowing firm conclusions about frequencies, at least in populations with similar ethnicity and healthcare access.

Interestingly, we found that younger patients with SLE seem to have an increased rate of AMI after long disease duration. Older patients, on the contrary, experienced AMI, shortly after receiving their SLE diagnosis and their rate was not increased compared with the population. Organ damage was reported to be associated with increased CVD risk,^27^ and we found that the accumulated organ damage was higher before first events, both for AMI and CVE, than among patients with SLE without such events. Noteworthy, the SLICC/ACR-DI is constructed without attribution, why other causes than SLE disease activity leading to damage might be of more importance in older patients getting AMI or CVE.

Both classical risk factors for CVD in SLE, such as hypertension, smoking, obesity, hyperlipidaemia and immobility, and SLE-specific risk factors have been proposed in the literature,^17^ and the prevalence of standard CVD risk factors was in fact higher in patients with SLE compared with healthy controls.^28–30^ In this series, hypertension and smoking at diagnosis were associated with events, smoking only for AMI and hypertension for both AMI and CVE. Of special interest was our finding that hypertension at diagnosis was associated with AMI only in patients diagnosed with SLE before the age of 55 years, which stresses the importance of blood pressure measurement in the clinical examination also of younger patients with SLE. The impact of other risk factors at diagnosis was not analysed in this series due to lack of information. Analysing risk factors over time was beyond the scope of this study.

Regarding SLE-associated risk factors the presence of anticardiolipin IgG antibodies was increased in patients with SLE and CVE in our study. This finding was not surprising; in fact we reported the association almost 30 years ago,^14^ and it has been confirmed repeatedly since then and others have shown that it was most pronounced when two or more antibodies were present.^31^

Several recent studies point out lupus nephritis as a risk factor for CVD. In a Swedish case–control study, excess atherosclerosis was seen in the SLE subgroup with nephritis as compared with the non-nephritis patients with SLE.^32^ Furthermore, in a recent nationwide Danish study, patients with lupus nephritis displayed an increased frequency of AMI.^18^ In parallel, our study presents data suggesting that reduced glomerular filtration rate below 50 mL/min is associated with AMI. This renal damage could be a consequence of disease activity, especially in younger patients with SLE getting AMI, since active lupus nephritis is more common in younger patients.^33^ However, since the DI is without attribution other causes of low glomerular filtration rate cannot be ruled out. We could not reproduce a recent finding of diabetes as a risk factor for cardiovascular events in SLE,^16^ probably due to limited number of diabetes cases in our cohort.

In recent publications from Canada AMI was analysed from registries of SLE cases and one finding was that AMI occurred early in the disorder and could even occur before diagnosis of SLE.^11 13^ Our results indicated more of a bimodal frequency of AMI, with the early events in older patients and the later events in patients diagnosed at a younger age. This pattern was not possible to detect in the western Canadian study since the follow-up was too short. We have not specifically addressed that AMI might occur even before the diagnosis of SLE, and in fact no such events were seen in the cohort used for IRR calculation.

Noteworthy, possible limitations in our study are time-dependent changes such as therapeutic improvements possibly affecting the outcome. In parallel, the development of better diagnostic modalities, for example, more advanced computer-analysed electrocardiography, MRI of the brain (generally available from 1990 in our hospital and partly replacing the more restrict used cerebral angiography) and new cardiac enzyme tests allowing more specific diagnosis of AMI. A thorough review of the clinical presentations in medical records by one of the authors (RFI) could to some extent compensate for these changes in diagnostic accuracy. However, these diagnostic and therapeutic changes also affect the population and thus do not influence the IRR calculations. Another limitation was that the numbers needed for statistical analysis are not sufficient for separate analysis of subarachnoid haemorrhages or intracerebral bleedings, but since only hypertension was retained in the multivariable analysis and hypertension certainly is a risk factor for bleeding we decided to handle all CVE together. Lack of information on certain risk factors at diagnosis was another limitation.

In conclusion, cardiovascular events are increased in SLE and are associated with hypertension, smoking and increased damage rate. This indicates that preventive measures from diagnosis should include good control of blood pressure, smoking cessation and, in addition to controlling disease activity, therapy adjustments to reduce the damage rate.
