# Correction: Disease duration, age at diagnosis and organ damage are important factors for cardiovascular disease in SLE

**DOI:** 10.1136/lupus-2020-000398corr1

**Published:** 2020-07-09

Nived O, Ingvarsson RF, Jöud A, *et al*. Disease duration, age at diagnosis and organ damage are important factors for cardiovascular disease in SLE. *Lupus Sci Med* 2020;7:e000398. doi: 10.1136/lupus-2020-000398

The article has been corrected since it was published online. In the published version, the co-authors’ names were misspelled as Ragnar Frey Ingvarsson and Helena Tyden. The correct names have been amended as Ragnar Freyr Ingvarsson and Helena Tydén.

