# Supplementary figures and images for: Disease duration, age at diagnosis and organ damage are important factors for cardiovascular disease in SLE
Source: Lupus Sci Med. 2020 Jun 24;7(1):e000398. doi: 10.1136/lupus-2020-000398 (PMC7319716; doi:10.1136/lupus-2020-000398)

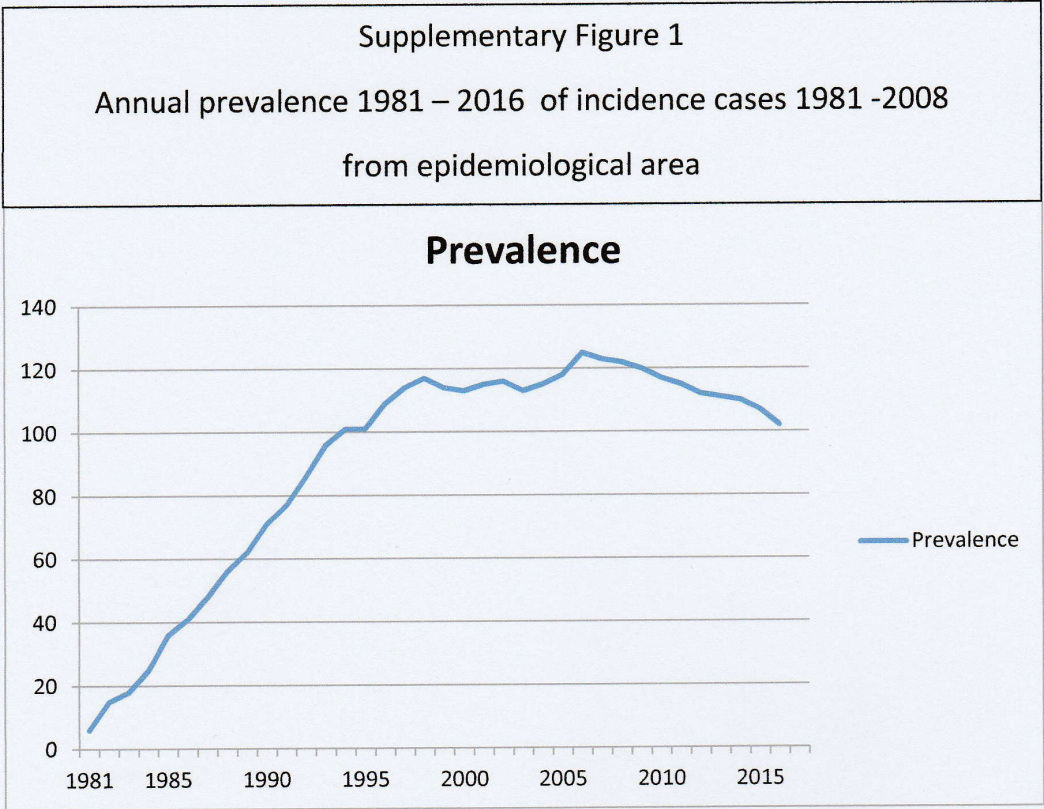

Supplement: Supplementary data [file lupus-2020-000398supp001.pdf]

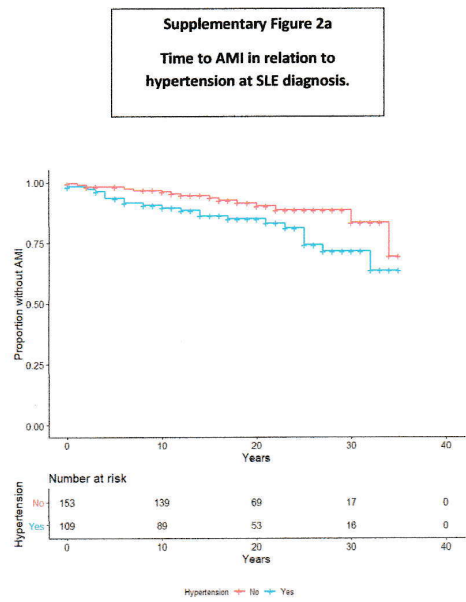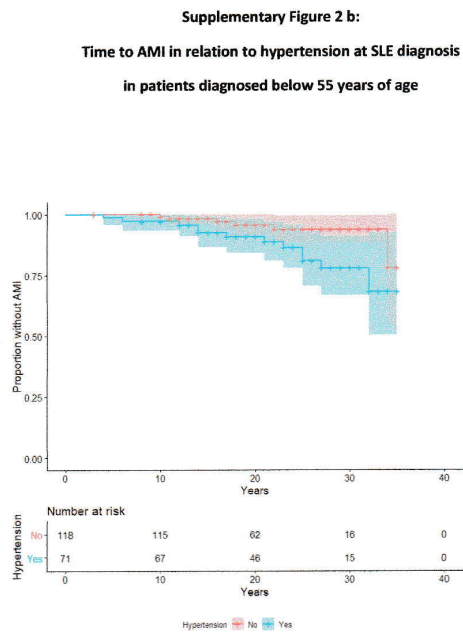

Log rank test:  $p=0.04$

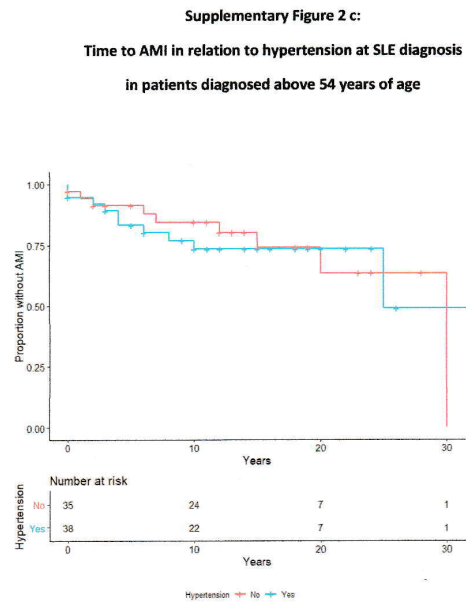

Log rank test:  $p=0.9$

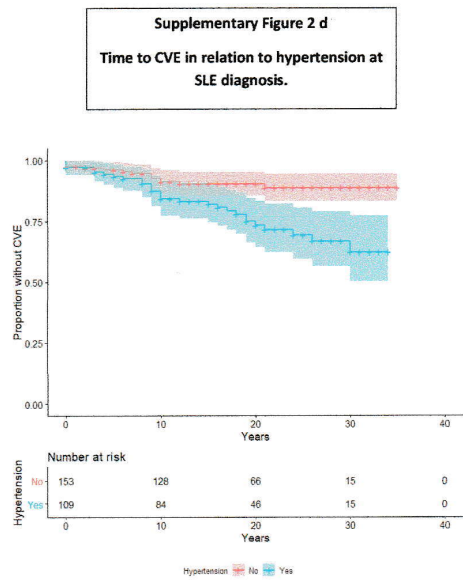

Supplement: Supplementary data [file lupus-2020-000398supp002.pdf]
